# Choledochal Cysts in Adults: A Case Report and Literature Review

**DOI:** 10.7759/cureus.72456

**Published:** 2024-10-26

**Authors:** Pablo Villarino Zapata, Elizabeth D Gutiérrez Cantón, Gilberto Samaniego Arvirzu, Cristobal A Aguilar Sibilla

**Affiliations:** 1 General Surgery, Hospital Regional de Alta Especialidad Dr. Gustavo A. Rovirosa Pérez, Villahermosa, MEX; 2 General Surgery and Gastrointestinal Endoscopy, Hospital Regional de Alta Especialidad Dr. Gustavo A. Rovirosa Pérez, Villahermosa, MEX

**Keywords:** acute cholangitis, choledochal cyst excision, choledochal cysts, roux-en-y hepaticojejunostomy, type 1a cyst

## Abstract

Choledochal cysts are a rare malformation of the biliary tract with an unknown etiology, predominantly affecting Asians and females. Although they are more often diagnosed during childhood, symptoms typically present in young adulthood due to complications. There are no pathognomonic clinical manifestations; the clinical presentation is associated with gallstones, choledocholithiasis, pancreatitis, cholangitis, and an increased risk of malignancy. We report a rare case of a choledochal cyst in adulthood, with delayed diagnosis and management due to the COVID-19 pandemic.

A 21-year-old woman, at 34.2 weeks of pregnancy, was diagnosed with cholangitis in 2022. She initially received minimally invasive treatment at another hospital but lost follow-up after discharge due to the COVID-19 pandemic. A year later, her family doctor referred her to our clinic, following protocol for gallstone disease.

A diagnostic protocol was initiated, involving multiple imaging studies such as ultrasound, endoscopic retrograde cholangiopancreatography (ERCP), and abdominal computed tomography (CT). These studies allowed us to identify and classify the cyst, enabling proper surgical planning. Open cyst excision with Roux-en-Y hepaticojejunostomy was performed.

Biliary cysts represent a rare pathology with potentially serious complications. They are not usually suspected at first in young adults, and the clinical presentation reflects both the cyst and its associated complications. A combination of clinical data, imaging studies, and endoscopy is essential for precise diagnosis, proper classification, and appropriate surgical planning. Total excision with biliodigestive reconstruction through Roux-en-Y bilioenteric anastomosis remains the preferred treatment. Long-term follow-up is necessary due to the potential for complications.

## Introduction

Biliary cysts are single or multiple dilatations, originally named choledochal cysts, as the most commonly involved segment is the common bile duct. They are considered congenital malformations, presenting with variable dilatation of the biliary tree, involving both intrahepatic and extrahepatic regions, including the intrapancreatic portion.

This condition is more frequent in females, with a female-to-male ratio of 3:1. Around 25% of cases can be diagnosed prenatally [1]. Biliary cysts are most commonly diagnosed during childhood and adolescence, with up to 80% of cases identified during these stages, while the remaining cases are diagnosed in adulthood [2].

Although the exact etiology remains unknown, two theories have been proposed. The first suggests that the cysts result from congenital stenosis of the biliary tract. However, the most widely accepted theory is the one proposed by Babbitt in 1969 [3], which associates the condition with an abnormal anatomical union between the bile duct and the pancreatic duct. According to this theory, an extended union (>15 mm) impairs the normal function of the common bile duct sphincter (Boyden sphincter), preventing it from blocking retrograde reflux of duodenal contents into the biliary tree. This reflux triggers a cycle of chronic inflammation, stasis, increased ductal pressure, dilatation, and cyst formation [4].

Biliary cysts are rare in the Western world, with an incidence of 1 to 2 cases per million people. In contrast, Asian countries, particularly Japan, report a much higher incidence of 1 in 13,000 births. The first documented case was reported by Halliday Douglas in 1852 [5], describing a 17-year-old female named Catherine, who presented with fever and jaundice. This illness does not have pathognomonic digestive symptoms but is often associated with gallstones, choledocholithiasis, cholangitis, pancreatitis, portal hypertension, and even malignant neoplasms. We present a rare case of a young pregnant woman diagnosed with cholangitis due to a choledochal cyst, with delayed diagnosis and treatment resulting from the COVID-19 pandemic.

## Case presentation

The patient first experienced symptoms in May 2021, at the age of 19 and 34.2 weeks pregnant. She was referred from a community hospital to the state capital, where she was initially evaluated at a second-level gynecology and obstetrics hospital. Due to the COVID-19 pandemic and the non-obstetric nature of her emergency, she was transferred to our specialty hospital, which provided obstetric care. The patient presented with clinical features including right upper quadrant abdominal pain, fever, and jaundice. On physical examination, she exhibited jaundice without signs of acute abdomen. A gynecologist assessed her and confirmed fetal-maternal wellbeing.

Laboratory tests and abdominal ultrasound were performed. The results from this first admission indicated cholangitis, gallstones without cholecystitis, and fusiform dilation of the extrahepatic bile duct with stones (Table 1). A conservative medical approach was chosen, and endoscopic retrograde cholangiopancreatography (ERCP) with sphincterotomy was performed, leading to pain relief and resolution of jaundice. The patient was discharged and returned to her community hospital, where she successfully completed her pregnancy.

**Table 1:** Laboratory Tests

Parameters	First Admission Laboratory Values	Reference Ranges
Hemoglobin (g/dL)	12.3	12–15
Hematocrit (%)	35.1	36–45
White blood count (10^3^/µL)	7.7	4.50–11
Neutrophils (%)	62.2	45–70
Platelets (10^3^/µL)	212	150–450
C-reactive protein (mg/L)	5	<10 mg/L
Prothrombin time (sec)	13.6	10–13
International normalized ratio (INR)	1.14	0.85–1.15
Amylase (U/L)	29.74	30–110
Lypase (U/L)	16.6	23–300
Glucose (mg/dL)	112	70–110
Creatinine (mg/dL)	0.4	0.60–1.10
Bilirubin-total (mg/dL)	0.3	0.20–1.10
Bilirubin-direct (mg/dL)	0.06	0.10–0.20
Bilirubin-indirect (mg/dL)	0.24	0.20–0.80
Alanine transaminase (IU/L)	21.63	10–42
Aspartate aminotransferase (IU/L)	23.38	10–60
Lactate dehydrogenase (IU/L)	177.27	90–180
Blood type	O, Rh-positive	–

Two years later, she returned to our specialty hospital. She was a young woman with no chronic illnesses, allergies, or prior surgeries, using a non-definitive fertility control method. Her only reported issue was food intolerance. On clinical examination, her vital signs were normal, and head, neck, and thorax examinations were unremarkable. The abdomen showed stretch marks, and Murphy’s sign was negative. An ultrasound from a private facility reported gallbladder stones. We proceeded with the case protocol, yielding the following results.

Abdominal ultrasound

The liver parenchyma appeared homogeneous, with generalized intrahepatic bile duct dilatation. The gallbladder measured 87 × 32 mm in longitudinal and transverse axes, with a wall thickness of 2 mm. It exhibited two kinks resembling a sigma shape and contained multiple stones, up to 11 mm in size, filling 80% of the gallbladder. The common bile duct could not be evaluated in its supraduodenal and intrapancreatic portions. The main pancreatic duct measured up to 2 mm. The portal vein had a diameter of 9 mm. The diagnostic conclusion included uncomplicated gallbladder disease with evidence of choledocholithiasis in the intraduodenal portion. The liver, pancreas, and spleen appeared sonographically normal.

Endoscopic retrograde cholangiopancreatography

**Figure 1:**
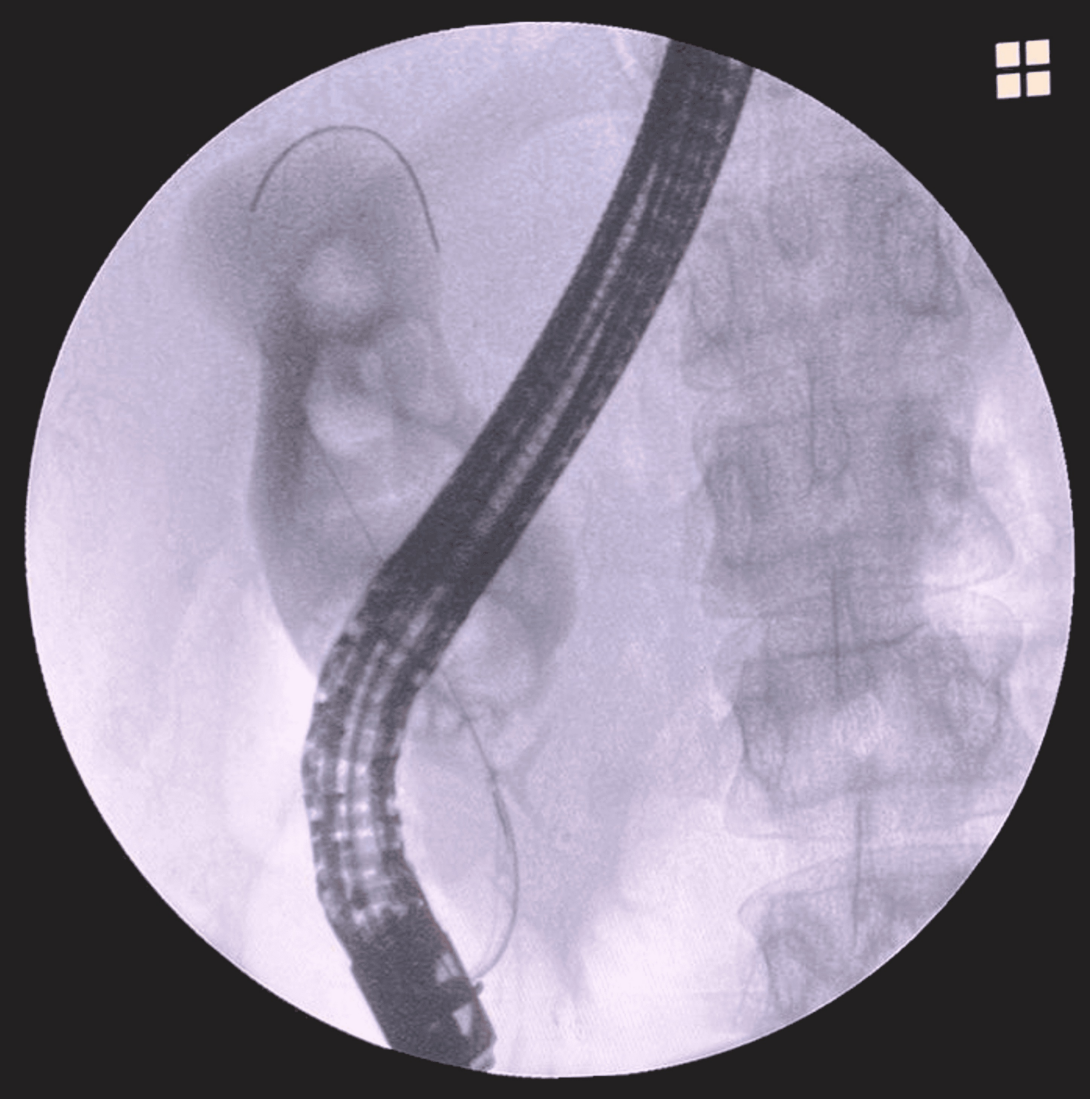
Endoscopic Retrograde Cholangiopancreatography The image shows a dilated common bile duct with contrast media, revealing the presence of multiple filling defects (stones within the choledochal cyst).

The major papilla appeared small, with post-papillotomy changes and spontaneous bile output. Cannulation was performed using a triple-lumen sphincterotome, and fluoroscopy confirmed the correct placement of the hydrophilic guide in the main bile duct, allowing for the injection of ionic water-soluble contrast. Multiple filling defects, suggestive of large stones measuring approximately 1 to 2.5 cm, were observed, along with significant dilation of the common bile duct and hepatic duct, both appearing fusiform (Figure 1). The cystic duct, gallbladder, and main pancreatic duct were not adequately opacified, and pancreatography was not performed. The diagnostic conclusion included fusiform dilation of the hepatic and common bile ducts (probable type IA vs. IC choledochal cyst) with unresolved choledocholithiasis.

Contrast-enhanced abdominal computed tomography

**Figure 2:**
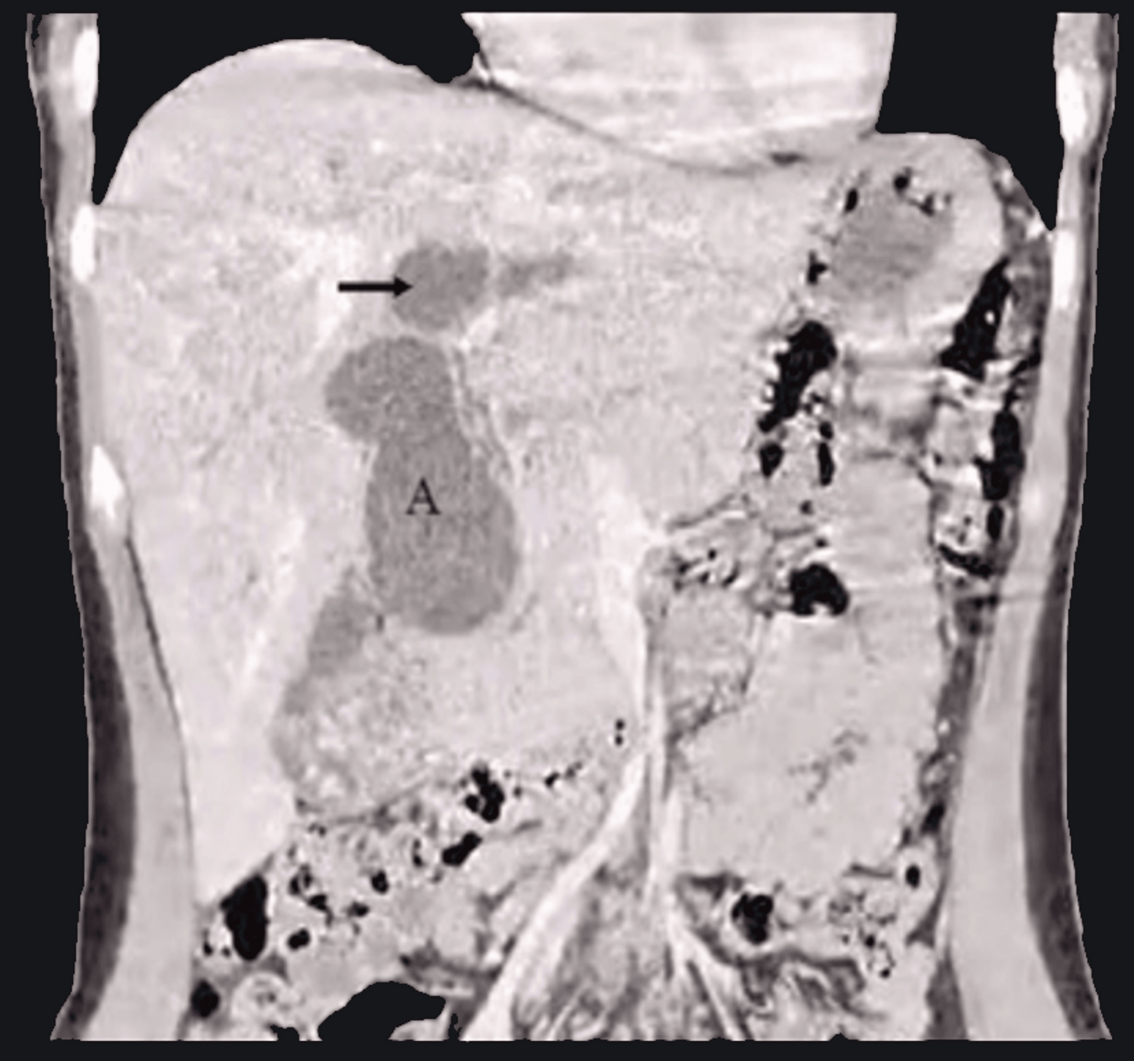
Contrast-Enhanced Abdominal Computed Tomography This coronal thoracoabdominal section shows both extrahepatic and intrahepatic bile ducts with significant dilation. The dilated common bile duct (A) is observed from its supraduodenal portion to the common hepatic duct (arrow), along with the left and right hepatic ducts.

The liver was in its usual position, with preserved morphology and size, and well-defined edges. The parenchyma exhibited appropriate echogenicity. There was dilation of the intrahepatic bile duct up to 10 mm. The gallbladder was in its usual position, with thickened walls measuring 3.7 mm, perivesicular edema, and heterogeneous content containing multiple well-defined hyperdense oval images. The common bile duct was distended throughout its course, with the intrapancreatic portion measuring up to 34 mm, containing multiple stones (Figure 2).

Magnetic resonance cholangiopancreatography

It is well known that magnetic resonance cholangiopancreatography (MRCP) should be used as standard diagnostic tool, as it will delineate anatomy and typing of choledochal cyst. But it was not performed due to the lack of resources in our hospital facility (MRI scanner).

Treatment and evolution

**Figure 3:**
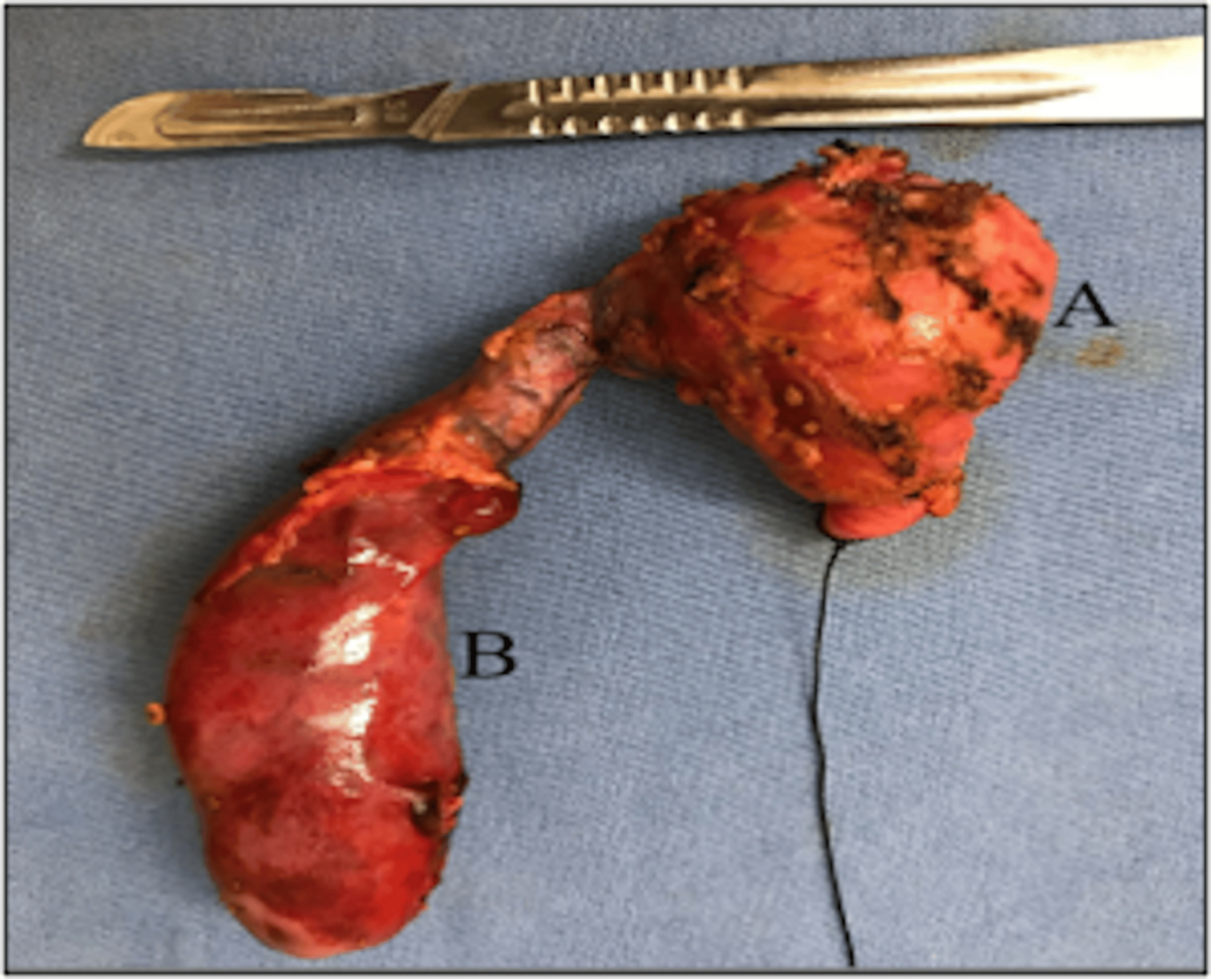
Photograph of Surgical Specimen Block resection of the common bile duct (A) along with the gallbladder (B). The common bile duct cyst measures 5 cm in its largest diameter. Gallblader measures are 7 x 3.2 cm.

The patient was prepared and scheduled for open cholecystectomy, resection of the choledochal cyst, and biliodigestive diversion in October 2023. The intraoperative findings were as follows: a folded gallbladder measuring approximately 70 × 30 mm with thin walls, containing multiple stones, and a short cystic duct. There was significant dilatation of the common bile duct throughout its length, with stones inside, classifying it as a Todani type 1-A cyst (Figure 3). A block resection of the gallbladder and cyst was performed. At the carina, two right hepatic ducts were identified, one normal-sized and one hypoplastic, along with a normal left hepatic duct. Both right hepatic ducts contained stones and biliary sludge. The stones were extracted, and thorough lavage was performed. Reconstruction of the biliary tract was carried out using a hepatojejunostomy in a Roux-en-Y configuration, in a single plane, with a drain left in place.

In recovery, the patient began oral intake 24 hours post-surgery, and the abdominal drain was removed on the seventh day. She developed a superficial surgical site infection, which was managed with monitoring, dressing changes, and antibiotic treatment until resolution. The patient was discharged on the thirteenth day of hospitalization. At one year of follow-up, she reports no pain, is asymptomatic, and remains under lifelong surveillance. We will continue to monitor tumor markers during follow-up, specifically carcinoembryonic antigen (CEA) and cancer antigen 19-9 (CA 19-9) serum levels, due to the risk of developing a metachronous biliary malignancy [6].

Histopathological report

The histopathological examination revealed a simple choledochal cyst and moderate acute-on-chronic cholecystitis with lithiasis. A paracystic hemangioma was identified, along with a cystic lymph node showing reactive mixed hyperplasia. There was no evidence of malignant neoplasia.

## Discussion

Choledochal cysts are most often diagnosed in childhood, but up to 20% of cases are identified in young adults. Diagnosing these cysts in children may be easier due to the classic triad of symptoms: abdominal pain, jaundice, and a palpable mass in the right upper quadrant. In adults, however, only about one-third exhibit this classic triad [2].

This condition is often discovered incidentally or when complications arise, such as ductal stenosis, gallstones, choledocholithiasis, cholangitis, pancreatitis, spontaneous cyst rupture, secondary biliary cirrhosis, or malignancy [7]. In our patient’s case, this aligns with the literature, as she presented in young adulthood with chronic gallbladder disease and cholangitis secondary to choledocholithiasis.

A high level of diagnostic suspicion is essential to prevent delays in diagnosis and avoid potentially severe complications. During the initial evaluation, the patient, a young pregnant woman, presented with symptoms that did not immediately suggest a choledochal cyst. Non-invasive imaging studies, such as ultrasound, played a crucial role in diagnosis. Her cholangitis was managed with ERCP, allowing her to complete her pregnancy without further complications.

In the second stage of care, the patient was asymptomatic, enabling us to continue with her evaluation. Laboratory studies returned normal results. We employed multiple imaging modalities-ultrasound, CT, and endoscopy. The ultrasound conducted at our hospital documented both gallbladder and choledochal pathology. A follow-up ERCP (Figure 1), a diagnostic and therapeutic tool with 100% diagnostic sensitivity and 90% specificity [8], confirmed the presence of a choledochal cyst.

An enhanced abdominal CT scan served as a complementary study, helping delineate the anatomy, classify the type of biliary cyst, and determine the appropriate surgical strategy.

It is noteworthy that the earliest description of this pathology was made by Douglas in 1852 [5], which later aided Alonso-Lej, Rever, and Pessagno in compiling 94 cases from the literature in 1959 to create the first clinical classification [9]. In 1977, Todani et al. proposed a new classification system, categorizing five subtypes based on the cyst’s location (Table 2) [10], although the underlying pathophysiology remains unclear.

**Table 2:** Biliary Cysts: Classification and Treatment Information obtained and modified from Todani's original classification and from UpToDate [10, 13].

Cyst Type and Presentation Rate	Description	Treatment
I (50-85%)	Fusiform dilatation of the common bile duct	Complete surgical excision of the cyst + Roux-en-Y hepatojejunum anastomosis
Ia	Fusiform dilatation of the entire common bile duct (common type)	
Ib	Segmental dilatation of the common bile duct	
Ic	Diffuse dilatation of the common bile duct	
II (2%)	Common bile duct diverticulum	Simple surgical excision
III (1-5%)	Saccular dilatation of the common bile duct in the ampulla of Vater, also called choledochocele	Endoscopic sphincterotomy; endoscopic loop resection
IV (15-35%)	Multiple dilatations of the common bile duct	Complete surgical excision of the cyst + Roux-en-Y hepatojejunum anastomosis
IV-A	Intra and extrahepatic cysts	
IV-B	Extrahepatic cysts only	
V (20%)	Fusiform or saccular cystic dilatation of intrahepatic bile ducts, associated or not with hepatic fibrosis (Caroli disease)	Supportive medical management for complications; liver transplantation

In recent years, modifications to Todani’s classification have introduced new categories to account for cystic duct dilation: type 1D or 6A for isolated cystic duct dilation, and 6B for dilation involving both the cystic and common bile ducts [11]. Additionally, studies have addressed the classification and management of distal biliary cysts located in the intrapancreatic portion of the common bile duct [12]. Our patient’s case was preoperatively classified as Todani type 1A.

The surgical goal is to remove all cystic tissue when possible due to the increased risk of malignancy associated with these cysts. A crucial aspect of the excision is recognizing that there is no clear distinction between a choledochal cyst and the adjacent normal bile duct; the resection margin is determined purely by morphology [13]. The inferior resection margins depend on the distal extent of the cyst, making preoperative imaging via ERCP, MRCP, endoscopic ultrasound, or intraoperative cholangiography essential for determining the appropriate margin [13,14].

During surgery, excising the cyst may be complicated by its proximity to surrounding structures. If dissection proves technically challenging, the literature suggests leaving the posterior wall of the cyst intact and performing a mucosectomy to reduce the risk of malignancy [15]. In our patient’s case, complete resection of the cyst up to the margin of the common hepatic duct was achieved.

Another challenge is reconstructing the biliary system through a Roux-en-Y hepatojejunostomy to facilitate biliary drainage from the liver. With advances in laparoscopic techniques and increasing surgical experience, many surgeons now perform this reconstruction laparoscopically. Although we followed established guidelines in treating our patient, we were unable to offer a laparoscopic approach due to limited resources. Nonetheless, our immediate results are comparable to those reported in the literature [16].

The most common long-term complication of hepaticojejunostomy is stenosis of the biliary-enteric anastomosis, which occurs in up to 30% of patients and may present as cholangitis, jaundice, or cirrhosis [13,17]. It is important to emphasize that cystic resection and biliodigestive diversion do not eliminate the risk of future malignancy. Therefore, patients must be monitored annually with liver function tests to detect potential complications. We will continue to follow this patient for life through our outpatient clinic.

## Conclusions

Biliary cysts are a rare pathology characterized by vague and nonspecific symptoms, often presenting with symptoms related to complications that can be potentially severe, such as gallbladder disease with or without cholecystitis, choledocholithiasis, cholangitis, pancreatitis, cirrhosis, and an increased risk of malignancy. While these cysts are more commonly diagnosed in childhood, diagnosis in young adults is rare, particularly among females.

Accurate identification of the cyst type and anatomical location is essential for planning appropriate surgical treatment. Complete resection of the cyst, followed by biliodigestive reconstruction via Roux-en-Y hepatojejunostomy in a single plane, is the ideal approach to ensure effective biliary drainage. Given the risk of biliary-enteric anastomosis stenosis and its potential complications, long-term follow-up is crucial.
